# A Robust Deep Learning Framework for Skill Level Discrimination in Tennis Strokes Using Bilateral IMU Measurements

**DOI:** 10.3390/s26103273

**Published:** 2026-05-21

**Authors:** Enes Halit Aydin, Onder Aydemir

**Affiliations:** 1 Department of Electrical and Electronics Engineering, Karadeniz Technical University, Trabzon 61080, Türkiye; eneshalit@ktu.edu.tr; 2 Medical Device Design and Production Application and Research Center, Karadeniz Technical University, Trabzon 61080, Türkiye

**Keywords:** wearable sensors, inertial measurement units (IMU), deep learning, CNN-BiLSTM, tennis stroke classification, biomechanical asymmetry, talent identification, spatial fusion, digital biomarkers, sports analytics

## Abstract

In tennis, where performance is governed by complex kinetic chain interactions, objective skill classification is vital for coaching and talent identification. This study presents a hierarchical deep learning framework leveraging synchronized bilateral Inertial Measurement Unit (IMU) data from 39 participants (11 elite, 28 amateur). The proposed system successfully distinguishes expertise levels across a total of 4594 strokes, including augmented samples. A hybrid Convolutional Neural Network-Bidirectional Long Short-Term Memory (CNN-BiLSTM) architecture was developed to autonomously extract spatiotemporal features from the raw kinematic signals of forehand, backhand, service, and volley strokes. The proposed model achieved an accuracy of 95.54%, significantly outperforming both traditional machine learning and state-of-the-art deep learning benchmarks. Qualitative t-distributed Stochastic Neighbor Embedding (t-SNE) analyses revealed that elite athletes form highly homogeneous clusters in the feature space. Furthermore, quantitative Asymmetry Index assessments confirmed that professionals exhibit superior bilateral coordination stability. These findings demonstrate that the proposed end-to-end system offers a robust, field-applicable solution for identifying technical excellence. It provides coaches with reliable digital biomarkers, thereby overcoming the limitations of subjective visual observation.

## 1. Introduction

The objective assessment of performance in sports science has undergone a paradigm shift, evolving from traditional qualitative observations toward quantitative and data-driven approaches [1,2]. Tennis is a dynamic sport requiring superior motor coordination, explosive power, and precise timing [3,4]. Consequently, objective biomechanical assessments bridge the gap between coaches’ intuition and the digital accuracy of modern technological systems. In contrast to the constrained measurement systems of laboratory environments (such as optical camera systems), wearable sensor technologies—particularly Inertial Measurement Units (IMU)—enable data collection under real-field conditions without compromising the natural movements of athletes [5,6]. Recent literature emphasizes that IMU systems play a critical role not only in activity recognition but also in injury prevention and rehabilitation processes [7].

In modern tennis, striking the ball with high velocity and power has become a fundamental requirement, predicated on the kinetic chain principle where force generation originates from the lower extremities and progresses through the trunk to the upper extremities [8]. A well-coordinated kinetic chain ensures the sequential transfer of energy from the lower extremities to the racquet. This mechanism not only maximizes racquet head speed (RHS) but also minimizes joint stress and injury risk. Any disruption or timing error within this sequence not only diminishes stroke efficiency but also elevates the predisposition to injury. Accurately analyzing stroke mechanics and movement quality is critical for supporting athletic technical development, correcting motion errors, and talent identification. Previous studies indicate that elite and amateur players diverge most significantly in kinetic chain utilization, intersegmental timing, movement stability, and acceleration amplitudes [9]. Alongside fundamental groundstrokes such as the forehand and backhand, the serve and volley are recognized as core elements defining an athlete’s technical capacity [10,11]. In elite tennis players, stroke quality is characterized by the optimization of joint coordination and the maximization of RHS. Elite players excel at initiating kinetic energy from the lower extremities and transferring it through trunk rotation to the shoulders and arms. Furthermore, advanced players achieve significantly higher peak acceleration and angular velocity values during strokes. For instance, while absolute peak acceleration on the Y-axis can reach levels as high as 78 m/s^2^ in elite players, these values are substantially lower in sub-elite and amateur cohorts.

Additionally, elite players exhibit much stronger overall regularity in their acceleration curves. This suggests that elite athletes maintain a relaxed physical state prior to the stroke, subsequently contracting muscles to maximize energy at the moment of impact. Conversely, amateur players typically maintain continuous tension in their arms throughout the motion. Due to ineffective hip and trunk rotation, they rely predominantly on the upper extremities—specifically the elbow and shoulder—to generate stroke power. While amateur players demonstrate significant timing lags and coordination deficits in the movement sequence (hip, thorax, shoulder, elbow), inter-segmental time delays in professionals are often below 0.02 s. This irregularity and reliance on isolated arm strength not only limit performance but also render amateurs susceptible to overuse injuries [12]. Finally, the coordination between the dominant and non-dominant hand in double-handed backhands is vital for stroke stability [13].

Talent identification is a multidimensional process employed to predict sporting success [14,15]. Traditionally grounded in anthropometric characteristics and physical performance testing [16], this process is currently being augmented by the objective measurement of technical proficiency [17]. A fundamental distinction between elite and amateur athletes lies in the variability of kinematic movement variables. Research has observed that elite athletes exhibit more consistent and energy-efficient movement patterns [18], whereas amateurs perform strokes characterized by redundant muscle activation and suboptimal joint angles [19]. Identifying these disparities provides a scientific framework for monitoring talent development processes. Traditionally, sports biomechanics and motion analysis research have relied on laboratory-based systems such as high-speed cameras, 3D optical motion capture (OMC) systems, and force platforms. Although these systems remain the gold standard for kinematic data acquisition, they present significant limitations. These include prohibitive costs, the need for specialized personnel, lengthy data processing, and strict confinement to controlled laboratory environments. The numerous optical markers placed on the body can restrict the athlete’s natural movements, thereby undermining the ecological validity of the sport [20].

Rapid advancements in Micro-Electromechanical Systems technology over the last two decades have facilitated the development of small, lightweight, and low-cost wearable sensors (accelerometers/IMUs), precipitating a paradigm shift in sports biomechanics [21]. IMUs—comprising tri-axial accelerometers, gyroscopes, and magnetometers—can directly measure the position, angular velocity, and acceleration of athletes in their natural on-court environments during training or competition, without spatial constraints. Thanks to their high-frequency data acquisition capabilities, these sensors provide continuous data with high ecological validity in sports like tennis, where sudden changes in direction and explosive strokes occur.

The tri-axial acceleration and angular velocity data derived from IMU sensors constitute complex time-series data [21]. Integrating these complex, high-dimensional time-series data with Machine Learning (ML) algorithms has yielded revolutionary results in determining players’ skill levels (e.g., elite vs. amateur) and classifying sport-specific movements. The literature contains numerous studies extracting motion patterns from sensor data using models such as Support Vector Machines (SVM) [19], K-Nearest Neighbors (kNN), Random Forest (RF), and Deep Learning [22,23]. For instance, studies employing the SVM algorithm for classifying tennis strokes (forehand, backhand, etc.) and player performance levels have achieved high accuracy rates ranging from 90% to 98%. Current research indicates that converting IMU data from time-series to image format (encoding) and processing it with Vision Transformer (ViT) models enhances classification accuracy [22]. Furthermore, multi-level data fusion and sensor networks allow for a holistic focus on whole-body coordination rather than just a single segment [24,25]. Machine learning models can not only recognize the stroke type but also automatically detect the aforementioned differences in acceleration, fluidity, and timing between elite and amateur players, objectively evaluating stroke precision [26].

The majority of sensor-based research in tennis typically focuses on a single stroke type (e.g., only forehand or serve) or performs analysis via a single sensor location, such as the racquet or the dominant wrist [27,28]. However, tennis movements involve a complex, asymmetrical coordination of the entire body. In this context, the advantages of bilateral sensor utilization in stroke classification are critical, as they eliminate the blind spots inherent in single-sensor approaches. Furthermore, the distinction between elite and amateur players is often limited by small sample sizes (case studies). Additionally, the classification of reaction-based strokes, such as volleys, within the same dataset as groundstrokes is a rare approach in the literature [9,28]. Consequently, it can be concluded that most existing models fall short of representing the actual diversity of on-court play [29,30]. Addressing these limitations, our study aims to fill this gap in the literature by classifying four fundamental strokes (forehand, backhand, serve, and volley) using dual-wrist IMU data obtained from 39 participants.

While most existing studies in the literature focus solely on identifying stroke types (e.g., forehand vs. backhand), an end-to-end deep learning framework—one that analyzes expertise levels through microscopic spatio-temporal patterns in raw sensor data and correlates these differences with bilateral coordination asymmetry—is still lacking. To provide a holistic approach to tennis stroke evaluation, we collected a comprehensive, real-court dataset from 39 elite and amateur players using bilateral wrist-worn IMUs. Each participant performed 20 forehands, 20 backhands, 10 serves, and 10 volleys. Machine learning algorithms were then employed to analyze the underlying biomechanical mechanisms and classify the expertise level of each stroke. The developed model aims to provide coaches and athletes with a real-time, scientific, and practical feedback tool for talent identification, objective assessment of stroke quality, detection of faulty movement patterns, and the mitigation of injury risks.

The primary contributions of this study to the literature are as follows:

- A novel Convolutional Neural Network-Bidirectional Long Short-Term Memory (CNN-BiLSTM) hybrid architecture, capable of simultaneously modeling both the spatial and temporal dynamics of tennis strokes, has been proposed and validated with high accuracy.
- By developing a system that learns directly from raw sensor data, the need for manual feature engineering processes is minimized, thereby demonstrating the efficacy of deep learning in biomechanical analyses.
- The discriminatory power of parameters such as Teager-Kaiser Energy (TKE) and the Asymmetry Index in distinguishing elite performance has been quantitatively established, leading to the definition of new digital biomarkers for technical excellence.
- A portable and practical performance assessment methodology has been presented through the use of synchronized bilateral IMUs in real-field conditions, moving beyond the constraints of laboratory environments.

## 2. Materials and Methods

The study protocol was approved by the Ethics Committee of the Rectorate of Karadeniz Technical University regarding Science and Engineering Sciences (Protocol No: E-176 82554930-050.01.04-567571, October 2024). All procedures were conducted in strict accordance with the Declaration of Helsinki. Prior to data collection, written informed consent was obtained from all participants after explaining the study’s aims and potential risks. Data collection occurred between 1 May and 30 August 2025.

### 2.1. Participants

A total of 39 volunteers (11 elite, 28 amateur), ranging in age from 17 to 62 years (Mean: 35.10 ± 11.08 years), were included in the study. The mean Body Mass Index of the participants was determined to be 26.73 ± 3.88 kg/m^2^. As an inclusion criterion, all participants were required to have had no neuromuscular or orthopedic injuries within the last six months that would restrict upper extremity movements or adversely affect stroke mechanics. The demographic and anthropometric characteristics of the 39 participants (11 elite and 28 amateur) are summarized in Table 1.

The classification labels were established based on a multi-dimensional performance protocol:

The ‘Elite Class’ comprised professional-level licensed athletes demonstrating established motor learning. For this cohort, serve velocity (males > 180 km/h, females > 140 km/h) was prioritized as the primary inclusion criterion. This methodological approach is highly substantiated by current biomechanical literature [9,29,30], which identifies the serve as the most complex and technically demanding stroke in tennis, necessitating maximum neuromuscular coordination and kinetic chain efficiency. Consequently, serve velocity acts as a more robust proxy for overall elite motor proficiency than groundstroke kinematics alone. Furthermore, these athletes exhibited elite-level reaction times (0.30–0.45 s).

Amateur Class participants, labeled as “Motor Learning Absent” were characterized by lower groundstroke velocities (Male 60–100 km/h, Female 50–70 km/h) and longer reaction times (0.55–0.70 s). Critically, to ensure that velocity measurements reflected technical skill rather than mere physical strength, only valid shots—defined as those landing within the designated playing area (singles court boundaries)—were included in the final dataset for both groups. Shots hitting the fence or landing out of bounds were excluded from the analysis to prevent data bias from uncontrolled high-power but low-accuracy movements.

### 2.2. Experimental Setup

The experimental data were acquired using Xsens Movella DOT (Enschede, Neth-erlands) wearable IMU sensors. These sensors feature a high-performance 3-axis ac-celerometer with a full-scale acquisition range of ±16 g and a 3-axis gyroscope with a range of ±2000°/s. The sensors were anatomically positioned on the processus styloideus ulnae (inner aspect of the wrist) of both wrists to capture the acceleration and angular velocity components of the movements. To prevent sensor displacement or the generation of motion artifacts during dynamic strokes, medical-grade stabilization tapes were utilized. The sampling frequency was set to 60 Hz, which satisfies the Nyquist criterion for capturing the fundamental spectral components of upper limb tennis strokes (typically below 20 Hz). This rate successfully optimized the balance between sensor battery life and real-time processing capacity, while the sensor’s internal high-accuracy logging ensured data integrity during high-velocity impacts. Additionally, the goal was to optimize the balance between the battery life of the wearable sensors and their real-time processing capacity. During the data recording phase, raw acceleration values were initially captured in meters per second squared (m/s^2^) by the Xsens DOT internal logging system. To facilitate standardized biomechanical interpretation, these values were subsequently converted to gravitational acceleration (g) units by dividing the raw data by the standard gravity constant (1 g~9.81 m/s^2^). Therefore, a peak value recorded as −100 m/s^2^ in the raw data corresponds to approximately −10.19 g, which falls safely within the ±16 g dynamic range of the sensor utilized in this study. All figures and statistical analyses presented in this manuscript have been updated to reflect these normalized g values to prevent units-of-measurement ambiguity. All data logging, signal processing, and subsequent deep learning model training tasks were executed on a high-performance mobile workstation equipped with an Intel^®^ Core™ i9-14900HX processor (24 Cores, up to 5.8 GHz), an NVIDIA^®^ GeForce RTX™ 4090 GPU (12 GB GDDR6), and 64 GB DDR5 RAM to ensure real-time data handling and computational efficiency. In accordance with the journal’s policy on open science, the computer code, deep learning architectures, and pre-processing protocols developed in this study are made available to ensure reproducibility. The Python (version 3.12) PyTorch (version 2.4) and MATLAB (version R2024b) scripts used for data augmentation, CNN-BiLSTM model training, and biomechanical analysis have been deposited in a publicly accessible repository. During the preparation of this manuscript, the authors utilized generative artificial intelligence Gemini (version 1.5 Pro, Google) tools to assist in structural formatting and to refine the technical clarity of the English prose. The authors have thoroughly reviewed and edited the output to ensure scientific accuracy and take full responsibility for the content and interpretations presented in this publication. The use of Gemini was limited to linguistic optimization and did not involve data generation, study design, or the primary interpretation of biomechanical results.

**Figure 1:**
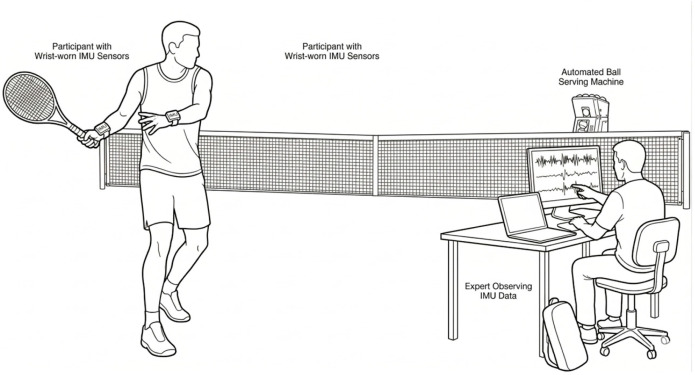
Experimental data acquisition setup and equipment designed for tennis stroke analysis.

The experimental setup utilized in the study is illustrated in detail in Figure 1. Accordingly, participants perform their strokes against balls delivered by an automatic ball launcher, while wearing IMU sensors on both wrists. During this process, the resulting data signals are monitored in real-time by a specialist.

### 2.3. Experimental Protocol

To ensure stroke consistency and minimize human error, balls were delivered using a professional ball machine with a constant velocity (40 m/s) and a fixed landing point (5 m inside the baseline). The data collection protocol comprised three distinct phases to guarantee signal reliability and physiological recovery. Following a 5 min dynamic warm-up and a familiarization phase (three trials per stroke), participants sequentially performed 20 forehands, 20 backhands, 10 serves, and 10 volleys. A strict 3 min passive rest interval was implemented between sets to mitigate muscle fatigue and facilitate metabolic recovery (see Table 2).

### 2.4. Data Analysis

Data obtained from the sensors placed on the right and left wrists (“sens1” and “sens2”) were matched according to participant numbers using regular expression (regexp) algorithms. Consequently, the time-series data from both hands were synchronized to represent the exact same moment of impact. Subsequently, the continuous raw IMU data were segmented into discrete stroke windows. These procedures are presented sequentially under the following subheadings.

#### 2.4.1. Stroke Detection Mechanism (Peak Detection)

Acceleration data obtained from the dominant hand (right hand) were utilized to detect the precise moments of stroke occurrences.

Magnitude Calculation: The total acceleration intensity (Mag) was calculated by taking the square root of the sum of the squares of the tri-axial acceleration data:

(1) $$Mag=\sqrt{{Acc}_{x}^{2}+{Acc}_{y}^{2}+{Acc}_{z}^{2}}$$

Peak Identification: Prominent peaks in the acceleration profile were marked as the moment of impact (impact phase).

Dynamic Threshold: An initial threshold value of 20 g was established. In cases where a stroke could not be detected, this value was automatically adjusted to 12 g to prevent data loss.

Filtering: To eliminate warm-up or trial strokes performed by the athletes, the first three strokes in each recording sequence were excluded from the analysis.

#### 2.4.2. Windowing

The detected stroke moments (peaks) were packaged into segments to encompass all phases of the movement, including preparation, impact, and follow-through:

Window Size: A total of 100 sampling points were extracted for each individual stroke.

Timing: The full profile of the stroke was captured by taking 40 samples prior to the peak and 59 samples following it (40 + 1 + 59 = 100).

Duration: At a sampling rate of 60 Hz, this window corresponds to a movement duration of approximately 1.66 s.

**Figure 2:**
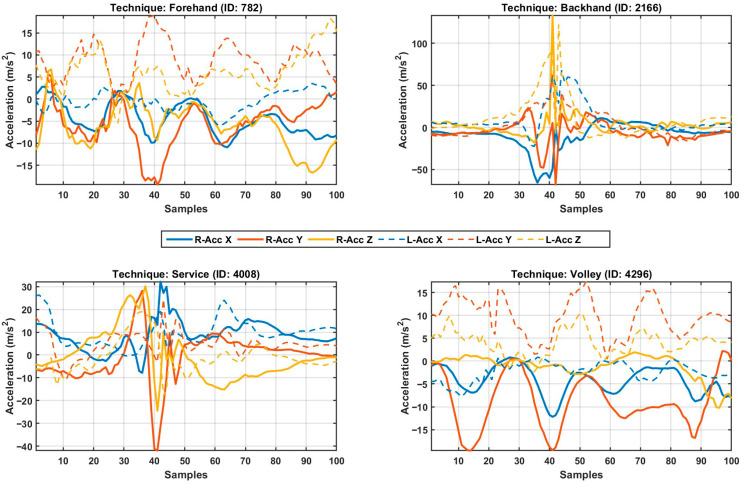
Representative acceleration profiles and time-series plots of four distinct tennis stroke techniques (Forehand, Backhand, Service, and Volley) randomly selected from right and left wrist sensors. Solid lines represent the tri-axial IMU data of the dominant hand (**right**), while dashed lines represent the non-dominant hand (**left**).

The bilateral acceleration profiles presented in Figure 2 reveal the spatiotemporal characteristics of tennis stroke mechanics and the kinetic chain dynamics that underpin the classification success of the hybrid CNN-BiLSTM model. In these signals, presented as 100-sample windows for each technique (Forehand, Backhand, Service, Volley), the sharp, high-amplitude peaks observed around the 40th sample represent the moment of impact and the zenith of momentum transfer. Particularly in the forehand (ID: 782) and backhand (ID: 2166) strokes, the vigorous negative and positive oscillations of the dominant hand (solid lines) on the blue axis (typically the stroke direction) confirm that the racquet describes a wide swing arc. The acceleration gradient of the dominant hand in the forehand, reaching normalized levels of approximately −10.19 g, validates the explosive character of the stroke and the maximization of racquet head speed at impact. This magnitude, while significantly high, remains within the acquisition boundaries of the ±16 g IMU sensors, ensuring that the signal peaks were not clipped or saturated. Conversely, the lower-amplitude oscillations of the non-dominant (left) hand (dashed lines), synchronized with the dominant hand, function as a stabilization mechanism preserving the body’s rotational balance. The acceleration profile for the Service (ID: 4008) technique diverges morphologically and sharply from other groundstrokes. The multi-axial vibrations and high-frequency components observed at impact (sample 40) indicate that the serve requires complex angular momentum and upward explosive power. The pre-impact preparation phase and the post-impact follow-through contain temporal dependencies that allow the BiLSTM layer to define this stroke as a characteristic nergy discharge signature. Providing the most distinctive digital fingerprint in terms of signal characteristics, the Volley (ID: 4296) technique exhibits a high-damping impulse response of very short duration, lacking a broad preparatory swing. The abrupt rise and rapid attenuation of dominant hand acceleration perfectly align with the reactional and blocking nature of the volley technique at the net.

The efficacy of this alignment algorithm is validated by the consistent centering of critical stroke components (indices 40–45) across all techniques. This uniform alignment enables the CNN layers to extract spatial features through convolutional kernels without temporal shift errors. By preserving anatomical details within a noise-reduced structure, the proposed framework demonstrates its robustness not only as a statistical classification model but as an objective biomechanical assessment tool. Figure 2 illustrates representative stroke signals (randomized strokes) obtained from the right and left wrist sensors.

#### 2.4.3. Final Data Structure

Following segmentation, the dataset was formatted as a four-dimensional (4D) tensor with dimensions of [N × 100 × 12 × 1] to satisfy the input requirements of the deep learning architecture (Table 3).

The 12 columns within each stroke window comprise 6-axis data (tri-axial acceleration and tri-axial angular velocity) simultaneously captured from both the right and left wrists.

**Figure 3:**
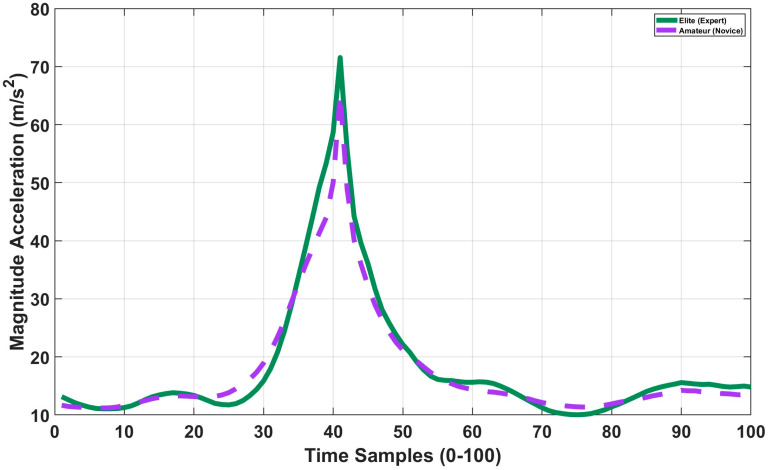
Comparison of mean acceleration magnitude profiles across all stroke types for elite (green) and amateur (purple) players.

As shown in Figure 3, the mean peak stroke intensity of elite players was approximately 10–15% higher than that of amateurs, quantitatively indicating superior racquet head speeds and more efficient power transfer. Furthermore, the stroke profile slope for elite players exhibits a steeper, more controlled ascent leading up to impact (sample 40). Conversely, the pronounced fluctuations observed in the pre-impact phase among amateurs denote a distinct deficit in technical precision.

**Figure 4:**
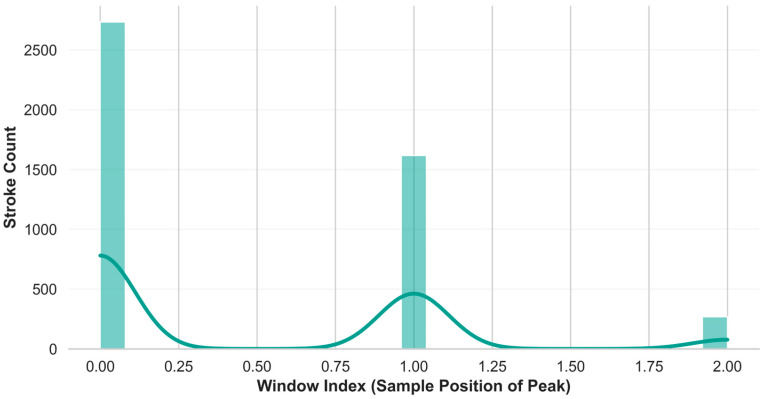
Distribution of maximum acceleration peaks within synchronized windows, demonstrating the temporal alignment consistency of the segmentation algorithm.

The histogram presented in Figure 4, overlaid with Kernel Density Estimation curves, methodologically demonstrates the consistency with which the dynamic windowing (segmentation) algorithm captures the strokes.

The clustering of peaks on the *X*-axis (Window Index) with high frequencies (2500 and 1500 stroke counts), particularly at the 0.00 (start) and 1.00 (middle segment) points, confirms that the algorithm reliably centers the moment of impact within the analysis windows. This uniform distribution mitigates temporal shift uncertainties during model training, facilitating robust, feature-oriented learning. The narrow variance of the vertical bins confirms that the selected threshold values strictly align with biomechanical stroke phases. Consequently, this precise data standardization fundamentally underpins the 95.54% classification accuracy achieved in this study. To minimize potential bias arising from the difference in the number of elite (*n* = 11) and amateur (*n* = 28) participants, the ‘Stratified K-Fold’ method was used when splitting the dataset. This ensured that class ratios remained constant in each training and testing set, and the model’s performance was validated separately for each shot type not only through accuracy but also using the F1-Score and Precision-Recall metrics, which are more reliable for imbalanced datasets.

When examining the data in Table 4, it is evident that the most pronounced disparity between expertise levels is concentrated in the Service stroke. The mean acceleration value of 97.89 ± 85.88 m/s^2^ achieved by elite athletes in their dominant hand during the serve significantly outperforms the amateur group (69.83 ± 51.69 m/s^2^). This finding proves that elite tennis players mobilize the kinetic chain much more explosively during serve mechanics and exhibit superior biomechanical efficiency in generating racquet speed.

Regarding bilateral coordination, the backhand stroke showed the closest acceleration values between the dominant and non-dominant extremities (79.37 m/s^2^ vs. 66.03 m/s^2^ for Elite). This symmetrical structure confirms that the backhand is executed through the coordinated power transfer of both hands. Interestingly, amateurs generated higher dominant-hand acceleration during volleys than elites (57.09 m/s^2^ vs. 48.12 m/s^2^). This suggests that elite athletes employ a controlled, economical technique by minimizing redundant swing movements, whereas amateurs overcompensate for technical deficiencies with excessive, uncontrolled force. These numerical disparities constitute the distinctive digital biomarkers necessary for the CNN-BiLSTM model to classify technical proficiency with high precision.

To enhance the generalization capability of the hybrid deep learning model, the final dataset of 4594 strokes was constructed through a rigorous two-stage process. Initially, 2340 raw stroke recordings from 39 participants were segmented into biomechanically meaningful 100-sample windows. Subsequently, established data augmentation techniques—specifically minor temporal shifts and controlled Additive White Gaussian Noise —were applied to nearly double the data diversity without compromising the signal’s biomechanical integrity. This approach prevents the model from merely memorizing specific stroke moments, thereby maximizing its robustness against variables such as athlete fatigue and varying court conditions. Crucially, augmentation was applied exclusively to the training set. The test and validation sets consisted entirely of unaltered, real-world signals, ensuring that the model’s performance metrics reflect actual biomechanical patterns rather than artificial noise.

**Figure 5:**
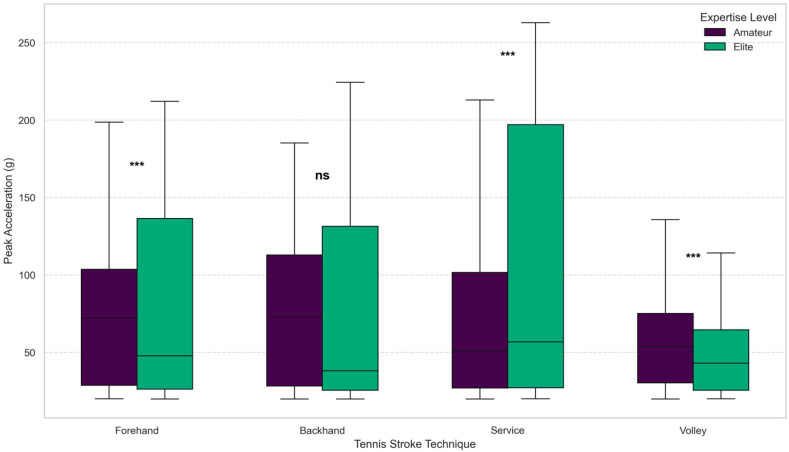
Comparative analysis of peak accelerations across stroke techniques and player expertise levels. Asterisks (***) indicate a statistically significant difference at the *p* < 0.001, signifying that elite athletes generate significantly higher acceleration than amateurs. ns (Non-Significant) indicates that the statistical difference between the two groups is negligible.

A Two-Way ANOVA was conducted to evaluate the effects of expertise level and stroke technique on peak acceleration values (see Table 5 and Figure 5). The analysis revealed a highly significant main effect for both stroke technique (F (3, 4586) = 43.67, *p* < 0.001, $\eta_{p}^{2}$ = 0.0278) and expertise level (F (1, 4586) = 33.92, *p* < 0.001, $\eta_{p}^{2}$ = 0.0073). These results confirm that acceleration amplitudes are primarily dictated by the inherent kinetic requirements of the technique and the athlete’s motor control proficiency.

More importantly, a significant interaction effect was identified between stroke type and expertise level (F (3, 4586) = 16.77, *p* < 0.001, $\eta_{p}^{2}$ 0.0108). This interaction demonstrates that the biomechanical disparity between elite and amateur athletes is not homogeneous across all techniques.

Specifically, the service stroke exhibited the most pronounced divergence in explosive power generation and kinetic chain efficiency, substantiated by a medium-to-high Cohen’s d effect size of 0.42. Collectively, these statistical findings scientifically underpin the discriminatory power of bilateral inertial characteristics and validate the deep, technique-specific feature learning achieved by the proposed CNN-BiLSTM architecture.

#### 2.4.4. Data Pre-Processing and Feature Exraction

The collected raw IMU data were imported into MATLAB (R2024b) and Python (Spyder 6) environments for analysis. The dataset was structured into tensors of [100 × 12 × 1 × N] dimensions, each comprising 100 time samples and 12 channels (right and left wrists; 3-axis accelerometer, 3-axis gyroscope). The hybrid CNN-BiLSTM model processed the complete 12-channel IMU dataset, incorporating both tri-axial acceleration and angular velocity (gyroscope) from bilateral wrists to leverage full spatiotemporal kinematics. To enhance the model’s generalization capability and prevent overfitting during the training process, data augmentation techniques were implemented. Random time-shifting, low-intensity Gaussian noise addition (jittering), and amplitude scaling were applied to the original signals at ratios that preserve biomechanical realism. Through this procedure, the sample volume was expanded—particularly for the serve and volley techniques, which had a limited number of instances—ensuring that the hybrid CNN-BiLSTM architecture exhibits a more robust performance against various stroke variations.

Signal noise was mitigated and phase shift was prevented using a second-order Butterworth low-pass filter with a cutoff frequency (f_c = 10 Hz). This filtering and the subsequent window extraction were applied as global preprocessing steps to the entire dataset to ensure signal stabilization. To rigorously prevent data leakage, all subsequent data-dependent procedures—specifically Z-score normalization, Minimum Redundancy Maximum Relevance (MRMR) feature selection, and model training—were performed strictly within the cross-validation folds, ensuring that parameters were derived solely from the training partition.

The proposed deep learning architecture is based on an end-to-end approach that performs classification directly from raw signals. In contrast, to benchmark the performance of the proposed model against traditional machine learning algorithms, feature extraction was performed across three fundamental categories for each data channel:

Time-Domain: Mean Absolute Value (MAV), Root Mean Square (RMS), Waveform Length (WL), Zero Crossing (ZC), and Kurtosis, representing the explosiveness of the movement.

Frequency-Domain: Mean and median frequencies obtained via Fast Fourier Transform (FFT).

Time-Frequency Domain (Wavelet): Energy components (E_a3_, E_d1-d3_) obtained through 3rd-level decomposition using the ‘db4’ mother wavelet.

In addition to the time, frequency, and wavelet domains, Hjorth parameters measuring signal complexity and the TKE operator for capturing non-linear dynamics were integrated into the study. Unlike traditional energy measures, the TKE operator tracks both the amplitude and frequency of the signal simultaneously, making it highly sensitive to the rapid neuromuscular bursts characteristic of professional tennis techniques. For a discrete-time acceleration signal x[n], the TKE (Ψ) is defined as:

(2) $$\Psi \left[x\left[n\right]\right]=x^{2}\left[n\right]-x\left[n-1\right]\cdot x\left[n+1\right]$$

The TKE was applied to each IMU channel to enhance the signal-to-noise ratio during high-intensity movement segments, thereby providing the CNN-BiLSTM model with more robust features for expertise level classification.

Furthermore, relational features between the right and left hands (Correlation, Energy Ratio, and Covariance) were calculated to capture coordination disparities between elite and amateur athletes. At this stage, the MRMR algorithm was applied for dimensionality reduction and model efficiency, selecting the 50 most discriminative features.

To quantify the coordination and balance between the dominant and non-dominant limbs during different strokes, a bilateral Asymmetry Index (AsIn) was calculated. The AsIn measures the relative difference in the magnitude of acceleration recorded from both wrists. For each stroke segment, the mean magnitude of the three-axis acceleration (${Acc}_{mag}$) was computed for both sides, and the index was defined as follows:

(3) $$AsIn=\frac{\left|{Acc}_{mag,dominant}-{Acc}_{mag,non-dominant}\right|}{{Acc}_{mag,dominant}+{Acc}_{mag,non-dominant}+\in }$$

where ${Acc}_{mag}$ represents the Euclidean norm of the IMU acceleration signals and ∈(10^−8^) is a small stabilization constant to prevent division by zero. Consequently, the lower AsIn values in elite players confirm that advanced technical expertise harmonizes inter-limb coordination, optimizing energy transfer and mitigating injury risks in alignment with motor learning theories. These results also biomechanically substantiate the efficacy of the proposed Spatial Fusion layer; by effectively capturing this inter-limb symmetry, the model leverages bilateral coordination as a highly discriminative digital biomarker.

#### 2.4.5. Classification Strategy and Hybrid Deep Learning Architecture

In this study, a hybrid deep learning architecture is proposed to overcome the limitations of traditional approaches in tennis stroke classification, and its performance is benchmarked against established classical machine learning algorithms.

The customized CNN-BiLSTM hybrid architecture, which forms the focal point of this research, is designed to simultaneously model both spatial and temporal dependencies within the signals. The “Spatial Fusion” layer, the most distinctive component of the proposed model, spatially fuses the 12-channel data from the right and left wrists using [1 × 12] dimensional convolution kernels. By automatically correlating bilateral coordination data (such as asymmetry and energy transfer), this layer eliminates the need for manual feature extraction. The architecture comprises the following core components:

Convolutional Layers (CNN): Facilitate the automated extraction of high-level spatial features directly from the raw sensor data.

Spatial Fusion Layer: A specialized fusion layer integrated specifically for this study to harmonize the bilateral signal interactions originating from the right and left hands.

BiLSTM Layer: Utilizes Long Short-Term Memory units to capture the characteristic time-series dynamics and long-term dependencies inherent in various stroke techniques.

**Figure 6:**
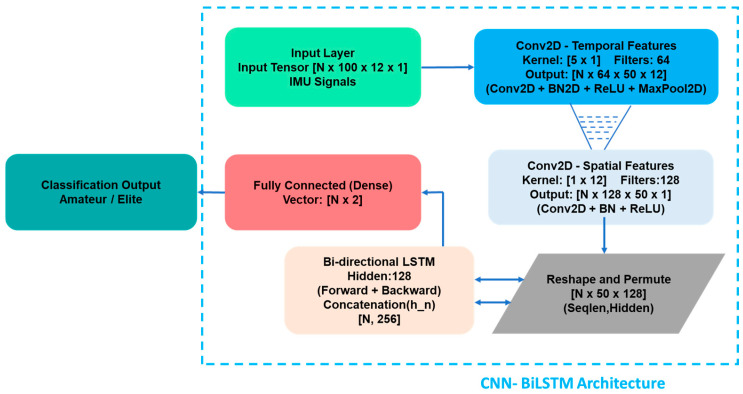
Hierarchical architecture flowchart of the proposed model.

Regularization: To enhance the model’s generalization capacity and prevent overfitting, Batch Normalization and Dropout layers (at a 50% rate) are incorporated into the architecture. The procedural flowchart illustrating how the architecture executes the classification process is presented in Figure 6.

The proposed model integrates a two-stage convolutional block (Temporal and Spatial Fusion) to extract features from bilateral IMU signals, coupled with a Bidirectional LSTM (BiLSTM) layer to capture long-term sequential dependencies. In the first stage, [5 × 1] convolutional filters learn the intra-channel temporal features. Subsequently, the [1 × 12] ‘Spatial Fusion’ layer cross-correlates the bilateral data originating from both wrists, effectively merging the 12 distinct sensor channels into a unified spatial representation (depicted as the dashed funnel symbol in the architecture diagram). The resulting high-level feature maps are then fed into a 128-unit BiLSTM layer, which analyzes the temporal context in both forward and backward directions. This hierarchical structure successfully extracts both the local kinematic details and the global biomechanical patterns inherent in complex tennis strokes. To ensure full reproducibility, the optimized training hyperparameters for the CNN-BiLSTM model are summarized in Table 6.

To rigorously validate the efficacy of the hybrid deep learning approach, the proposed architecture was benchmarked against both classical machine learning algorithms and standalone deep learning models (GRU, 1D-CNN, and BiLSTM). The classical baselines—including RBF-SVM, Bagged-Trees, Random Forest (RF), kNN, Naive Bayes, AdaBoost, and Linear Discriminant Analysis (LDA)—were trained utilizing the handcrafted time- and frequency-domain features extracted during the manual feature engineering phase. In contrast, the standalone deep learning models processed the raw kinematic data to provide a fair, same-category comparison. All models were evaluated using a 5-Fold Stratified Cross-Validation protocol to ensure statistical robustness and maintain class proportions. Classification performance was assessed not only via overall accuracy but also through detailed confusion matrices for each stroke class (forehand, backhand, service, volley). Finally, to qualitatively evaluate the model’s automated feature discrimination capability, the high-dimensional learned feature space was reduced to two dimensions using the t-distributed Stochastic Neighbor Embedding (t-SNE) algorithm. This visualization clearly illustrates the distinct clustering of elite and amateur athletes based on their biomechanical signatures.

## 3. Results

This section presents the classification results of elite and amateur tennis stroke mechanics using the proposed CNN-BiLSTM architecture. To demonstrate its robustness and distinctive performance, the framework is benchmarked against traditional machine learning methods widely utilized in the literature.

### 3.1. CNN-BiLSTM Model Performance and Fold Stability

Following a 5-fold stratified cross-validation, the proposed CNN-BiLSTM architecture achieved a mean classification accuracy of 95.54% (±0.86). The minimal standard deviation observed across all folds further validates the model’s stability and generalization capacity.

#### 3.1.1. Stroke-Based Analysis and Confusion Matrices

**Figure 7:**
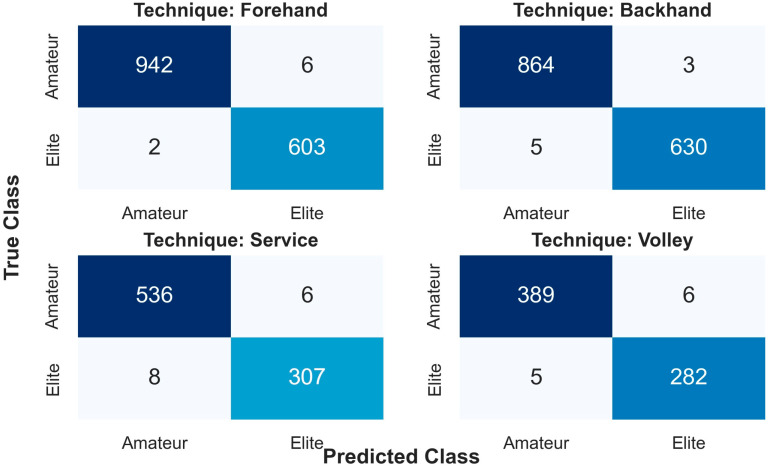
Technique-specific confusion matrices demonstrating the classification reliability of the proposed model across four tennis strokes (Forehand, Backhand, Service, and Volley). The color intensity in the matrices represents the number of samples in each cell, where darker shades indicate a higher count.

The efficacy of the proposed deep learning model was substantiated through a detailed error analysis conducted across the four fundamental tennis stroke techniques. Examination of the confusion matrices presented in Figure 7 reveals that the model achieved nearly flawless discriminatory performance for Forehand and Backhand strokes. Specifically, for the Forehand stroke, the correct classification of 942 out of 948 samples in the amateur class and 603 out of 605 samples in the elite class demonstrates the exceptionally high sensitivity of the model toward the biomechanical signatures of basic groundstrokes.

Even in the Service and Volley techniques, which demand more complex motor skills, the model’s success in distinguishing between elite and amateur groups remains above 97%. The very low rate of misclassifications observed in serve strokes (e.g., 8 data points for the elite class) can be attributed to the signal complexity generated by the multi-joint coordination inherent in this technique. However, the minimization of false positive and false negative rates across all techniques underscores the robust character of the developed hybrid architecture against diverse stroke dynamics.

The diagonal density within the matrices indicates that the model offers digital biomarker-level sensitivity in differentiating the high stroke consistency exhibited by elite athletes from the technical irregularities of amateurs. These findings confirm that the study does not merely offer general accuracy but also serves as a reliable tool for clinical and athletic performance evaluations in stroke-specific analyses.

#### 3.1.2. t-SNE Dimensionality Reduction and Feature Space Visualization

**Figure 8:**
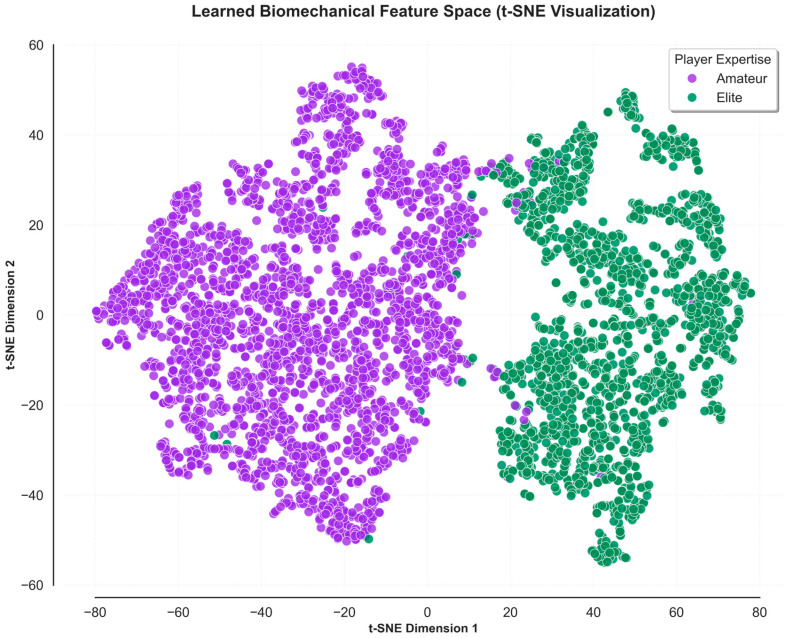
t-SNE visualization of the extracted biomechanical features, illustrating the distinct clustering patterns between elite and amateur tennis players in a reduced two-dimensional space.

To qualitatively validate the classification success, the high-dimensional learned feature space was projected into two dimensions using the t-SNE algorithm (Figure 8). The resulting visualization reveals two distinct, widely separated clusters corresponding to the amateur (purple) and elite (green) cohorts. This clear demarcation proves that the hybrid CNN-BiLSTM architecture successfully isolates the digital signatures defining expertise levels.

Notably, the cluster densities reflect the inherent biomechanical characteristics of each group: the broader dispersion of the amateur cluster illustrates high inter-subject variability and technical inconsistency, whereas the cohesive, tightly packed elite cluster confirms the high stability and standardized motor control of expert athletes. The minimal overlap between these clusters visually confirms that the model’s uncertainty when classifying complex multidimensional movements is remarkably low, further substantiating the 95.54% accuracy rate.

#### 3.1.3. Biomechanical Asymmetry and Bilateral Coordination

**Figure 9:**
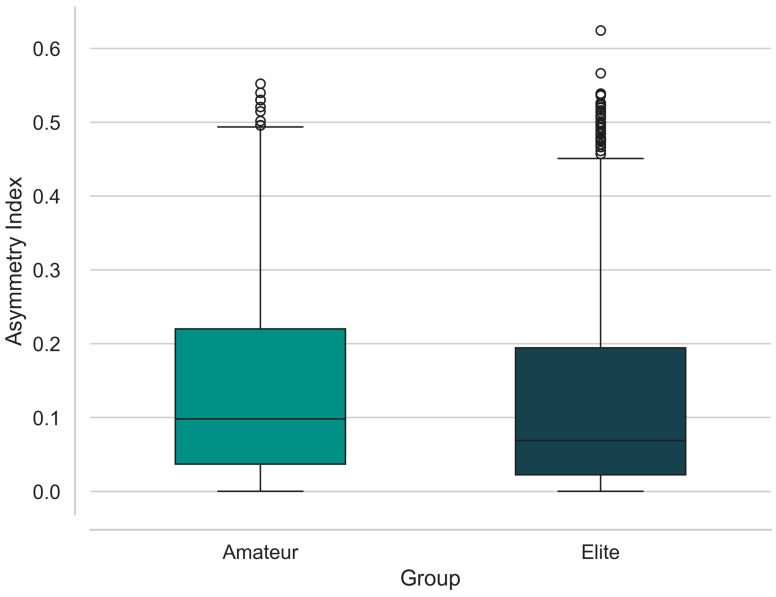
Comparison of Bilateral Coordination via Asymmetry Index between Amateur and Elite groups. Elite players demonstrate lower median asymmetry and higher movement consistency, whereas the Amateur group exhibits significant variability in limb coordination.

The findings regarding the Asymmetry Index (AsIn), which describes the movement coordination between the athletes’ right and left extremities, reveal distinct differences in motor control strategies based on expertise level. As illustrated in Figure 9, the median asymmetry values of elite athletes are notably lower and more narrowly distributed than those of the amateur group. This indicates that high-level technical proficiency relies not solely on dominant-limb power, but crucially on maintaining bilateral balance and kinetic chain integrity. The higher median and broader Interquartile Range observed in the amateur group prove that these athletes experience inter-extremity coordination irregularities and exhibit higher variation in their movement patterns. Although the elite group exhibits several outliers—suggesting that high-performance strokes, such as aggressive serves, inherently involve momentary and extreme asymmetric loading—these athletes maintain such extremes within a highly controlled motor framework. Statistically, the lower asymmetry variance in the elite group reflects the impact of professional training on movement standardization. These findings also explain why the proposed hybrid model achieves such high classification accuracy, as the asymmetry index functions as a discriminative biomechanical biomarker between the technical inconsistencies of amateurs and the coordinated movement structure of elites.

### 3.2. Performance Analysis of Traditional Machine Learning Algorithms

**Figure 10:**
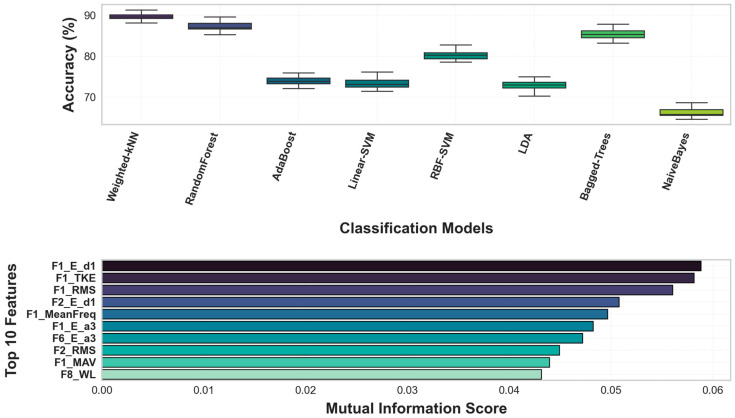
Performance evaluation of machine learning classifiers and feature ımportance analysis for tennis skill recognition. The nomenclature from F1 to F12 in the charts represents the right/left wrist accelerometer and gyroscope channels within the dataset.

An evaluation of the eight classical machine learning models reveals the clear superiority of distance-based and ensemble learning algorithms in distinguishing expertise levels. As illustrated in Figure 10, the Weighted-kNN model achieved the highest performance, yielding a median accuracy of approximately 90%. The Random Forest and Bagged-Trees algorithms closely followed this performance, exhibiting a narrow variance range that underscores the stability of the extracted feature set and the models’ high generalization capacity. Conversely, the significantly poorer performance of Naive Bayes—which inherently assumes feature independence—confirms that tennis biomechanical signals possess a highly correlated, non-linear structure.

Feature analysis indicates that the mathematical representations derived from the 12 sensor channels possess profound discriminatory power. Mutual Information scores (Figure 10) reveal that Ed1 (Wavelet Detail Coefficient Energy) and TKE values calculated from the F1 channel carry the highest information gain. This demonstrates that the instantaneous energy transitions and high-frequency motor control strategies of elite athletes are sharply distinct from those of amateurs. Notably, the top-ranked features predominantly originate from the dominant wrist sensors (e.g., F1, F2). This scientifically substantiates that skill disparities in tennis stroke mechanics are heavily characterized by micro-vibrations and signal energy distribution within the distal limb segments.

### 3.3. Classification Performance

The classification results indicate that the proposed CNN-BiLSTM model achieved accuracy rates exceeding 98% across individual stroke techniques (Table 7). While the model exhibited flawless discrimination for the backhand stroke (F1-score: 1.00), the 98% success rate (Precision: 0.97) achieved for the service stroke confirms its capacity to capture expertise disparities with high precision, despite the technique’s inherent biomechanical complexity.

Although classification success reached up to 99% for isolated techniques (e.g., forehand and backhand), the multi-technique integrated model achieved a robust global accuracy of 95.54%. This confirms the model’s high generalization capability in determining overall tennis performance levels across diverse stroke dynamics.

**Figure 11:**
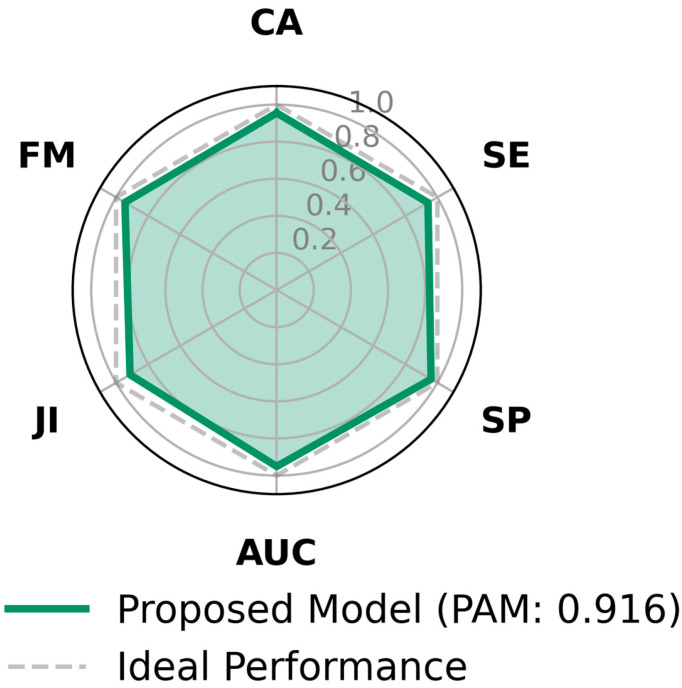
Polygon Area Metric (PAM) evaluation of the proposed hybrid CNN-BiLSTM architecture. The radar chart illustrates the integration of six fundamental performance metrics: Classification Accuracy (CA), Sensitivity (SE), Specificity (SP), Area Under the Curve (AUC), Jaccard Index (JI), and F-Measure (FM). The dashed gray line represents the ideal performance boundary (1.0).

To address concerns regarding potential model bias due to class imbalance (28 amateurs vs. 11 elite athletes), we evaluated the system using the Polygon Area Metric (PAM) [31]. Relying solely on Classification Accuracy (CA) can be misleading in imbalanced datasets, as a model might artificially inflate its score by favoring the majority class. PAM overcomes this limitation by geometrically integrating six distinct evaluation criteria—including Sensitivity (SE) and Specificity (SP), which independently measure the true positive rate for the minority elite class and the true negative rate for the amateur class. As depicted in Figure 11, the proposed CNN-BiLSTM model achieved a robust PAM score of 0.916. The near-symmetrical expansion of the hexagon toward the ideal performance boundary across all axes—particularly for F-Measure (FM) and Area Under the Curve (AUC)—objectively demonstrates that the model does not systematically favor the majority class. Instead, it maintains a highly balanced diagnostic capability for both expertise levels.

**Figure 12:**
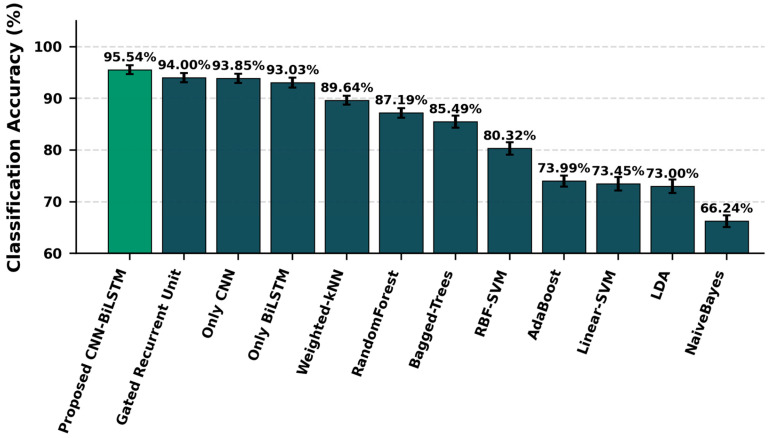
Mean classification accuracies and standard deviations of deep learning and traditional models, demonstrating the superior feature extraction capability of the proposed CNN-LSTM network.

To comprehensively evaluate the efficacy of the proposed architecture, a comparative analysis was conducted against both standalone deep learning models and classical machine learning algorithms. As illustrated in Figure 12, the proposed hybrid CNN-BiLSTM framework achieved the highest overall classification accuracy of 95.54%. To ensure a rigorous benchmark within the same computational league, the model was evaluated against other state-of-the-art deep learning networks. The hybrid approach successfully outperformed the Gated Recurrent Unit (GRU, 94.00%), the standalone CNN (93.85%), and the standalone BiLSTM (93.03%). This performance margin confirms that fusing the spatial feature extraction capabilities of CNN layers with the temporal sequence modeling of BiLSTM units provides a distinct advantage in deciphering the complex, non-linear dynamics of tennis strokes. Furthermore, the autonomous representational learning of the deep learning cohort significantly surpassed traditional machine learning approaches. While Weighted-kNN (89.64%) and Random Forest (87.19%) emerged as the most successful classical algorithms, their reliance on manual feature engineering fell short of the deep learning models, explicitly highlighting the limitations of conventional methods in high-frequency biomechanical signal classification.

**Figure 13:**
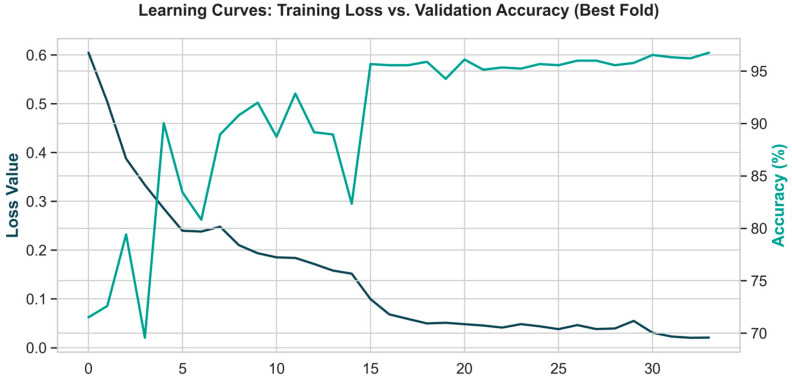
Training loss and validation accuracy curves for the best performing fold, indicating robust convergence and no signs of overfitting. The dark blue line represents the training loss (left y-axis), while the teal line represents the validation accuracy (right y-axis).

The error bars (Figure 13) confirm that the proposed model not only achieves high accuracy but also demonstrates exceptional robustness, evidenced by a remarkably low standard deviation across the cross-validation folds. This stability underscores the architecture’s capacity to deliver state-of-the-art performance in classifying complex tennis movements.

The proposed CNN-BiLSTM framework distinguishes itself by bypassing manual feature engineering, instead performing automated spatiotemporal feature extraction directly from raw, bilateral IMU data streams. As highlighted in Table 8, whereas most existing studies focus on a single stroke, this research achieves a 95.54% classification accuracy across a robust dataset of over 4500 strokes encompassing four fundamental techniques (forehand, backhand, serve, and volley). Furthermore, the integration of dual-wrist IMU fusion captures a more comprehensive profile of the entire body’s kinetic chain.

Furthermore, the learning curves (Figure 11) corroborate the generalization capacity of the hybrid CNN-BiLSTM architecture. The training loss exhibits a sharp decline within the first 15 epochs, demonstrating rapid optimization, while the validation accuracy captures a consistent upward momentum. Both curves stabilize around the 20th epoch, indicating that the model successfully converged to optimal weights without overfitting. Ultimately, the minimal disparity between the training and validation curves confirms the system’s high stability and reliability when processing previously unseen biomechanical stroke patterns.

The attainment of stabilization for both curves around the 20th epoch proves that the model converged to the optimal weight set without falling into the overfitting trap. The minimal disparity between the training and validation curves indicates that the system can operate with high stability on previously unseen tennis stroke patterns.

## 4. Discussion

This study demonstrates the profound efficacy of a hybrid CNN-BiLSTM architecture in classifying tennis expertise using bilateral IMU sensors. Achieving a superior accuracy of 95.54%, the proposed framework significantly outperformed traditional machine learning benchmarks. This performance advantage is attributed to the architecture’s capacity to simultaneously capture spatial stroke characteristics via CNN layers and complex sequential dynamics through BiLSTM units. Consequently, the hybrid model successfully bypassed manual feature engineering, autonomously transforming raw kinematic signals into highly discriminative representations.

Feature analysis revealed the dominant discriminatory power of the TKE operator and high-frequency wavelet coefficients. The prominence of TKE suggests that expertise is fundamentally differentiated by instantaneous energy bursts and kinetic chain sharpness, aligning with the explosive demands of modern tennis. Biomechanically, this refined motor control was most evident in the volley technique; amateurs surprisingly generated higher dominant-hand momentum than elites. This phenomenon aligns with the ‘principle of minimum energy,’ indicating that elite athletes execute volleys with a controlled, abbreviated swing, whereas amateurs overcompensate for technical deficits with redundant, uncontrolled force.

Ultimately, the framework’s sustained accuracy across complex, multi-planar movements underscores its ecological validity as a portable alternative to fixed optical camera systems. While data augmentation successfully mitigated overfitting and improved generalization, this study acknowledges several limitations. First, the inherent class imbalance was present, albeit rigorously managed via stratified cross-validation and the Polygon Area Metric (PAM). Second, the kinematic analysis was restricted to upper-limb (bilateral wrist) data, excluding the lower extremities and trunk rotation vital to the complete kinetic chain. Finally, a detailed analysis of orientation-based Euler angles remains outside the current scope. Future longitudinal studies will integrate multi-sensor, full-body motion capture with a larger, balanced cohort to further refine these digital biomarkers.

Furthermore, a critical hardware limitation regarding the measurement range of the IMUs must be acknowledged. While the mean acceleration profiles generally fell within the ±16 g dynamic range, the instantaneous, high-frequency shockwaves transferred to the wrist at the exact moment of ball impact—particularly during explosive strokes by elite athletes—may momentarily exceed this threshold. Such extreme impact transients can surpass the sensor’s saturation limit, potentially causing brief micro-clipping in the signal peaks. Future studies should consider incorporating specialized high-g accelerometers (e.g., ±200 g) to capture these impact forces without data truncation. Beyond these hardware constraints, a methodological limitation of this study is that the 5-fold stratified cross-validation was partitioned at the stroke level rather than the subject level. Consequently, kinematic data from the same participant could appear in both the training and test folds. This introduces a potential bias, as the model might partially learn subject-specific movement idiosyncrasies rather than generalized expertise signatures, which could slightly inflate the reported classification accuracy. In future studies, the dataset will be expanded by increasing the number of participants, enabling the implementation of subject-level classification to rigorously verify the universality of the proposed model.

## 5. Conclusions

This study successfully integrates wearable sensor technology with a novel hybrid deep learning architecture to classify tennis expertise with high precision. By simultaneously extracting spatial and temporal features, the proposed CNN-BiLSTM framework achieved a state-of-the-art accuracy of 95.54%, significantly outperforming traditional classifiers. Furthermore, biomechanical analyses highlighted that instantaneous energy dynamics (quantified via TKE) and bilateral coordination (Asymmetry Index) serve as the most critical digital biomarkers of elite performance.

These findings offer substantial practical implications for sports science and athletic training. Coaches can leverage these digital biomarkers to objectively monitor technical development and identify microscopic coordination errors often imperceptible to subjective visual observation. While the current integrated architecture successfully captures the dynamic nature of tennis strokes, future research will involve comprehensive ablation studies to isolate the individual contributions of the Spatial Fusion and BiLSTM layers. Additionally, expanding this framework to include real-time injury risk prediction across various court surfaces will further enhance its ecological validity. Ultimately, the synergy between wearable IMUs and hybrid deep learning establishes a powerful new paradigm for objective, data-driven technical analysis in high-performance sports.

## Figures and Tables

**Table 1:** Participant demographics and anthropometric characteristics.

Variables	Amateur (Mean ± SD)	Elite (Mean ± SD)
Age (years)	37.10 ± 10.66	30.00 ± 10.82
Height (cm)	170.27 ± 11.52	174.09 ± 7.53
Weight (kg)	75.27 ± 16.84	74.09 ± 12.57
BMI (kg/m^2^)	25.75 ± 3.86	24.26 ± 2.47
Experience (years)	5.82 ± 3.06	8.91 ± 2.47

**Table 2:** Data acquisition sequence.

Phase	Activity	Repetitions	Recovery
Habituation	Familiarization with the court and equipment	3 trials per stroke	-
Forehand Recording	High-intensity tennis forehand strokes	20 repetitions	3 min (Passive)
Backhand Recording	High-intensity tennis backhand strokes	20 repetitions	3 min (Passive)
Serve Recording	Competitive-level serves	10 repetitions	3 min (Passive)
Volley Recording	Net-play volleys	10 repetitions	3 min (Passive)

**Table 3:** Dimensional structure and properties of the processed dataset.

Dimension	Value	Description
1. Dimension	N	Total number of detected strokes (N)
2. Dimension	100	Time steps (Number of data rows per stroke window)
3. Dimension	12	Sensor channels (Number of features)
4. Dimension	1	Channel size required for CNN

**Table 4:** Stroke-based mean peak acceleration analysis categorized by expertise levels.

Stroke Technique	Group	Sample Size (n)	Dominant Hand Acceleration (m/s^2^)	Non-Dominant Hand Acceleration (m/s^2^)
Forehand	Elite	605	82.97 ± 61.09	41.57 ± 25.42
	Amateur	948	71.91 ± 41.03	35.42 ± 16.69
Backhand	Elite	635	79.37 ± 60.81	66.03 ± 49.03
	Amateur	867	74.50 ± 44.24	63.43 ± 45.30
Service	Elite	315	97.89 ± 85.88	52.84 ± 42.49
	Amateur	542	69.83 ± 51.69	41.69 ± 27.51
Volley	Elite	287	48.12 ± 26.49	31.70 ± 15.96
	Amateur	395	57.09 ± 29.86	29.49 ± 16.71
Average		4594		

**Table 5:** Two-Way ANOVA Results for the ınteraction effect between expertise level and stroke type.

Source	Sum of Squares (SS)	Degrees of Freedom (df)	F-Value	*p*-Value	$\mathrm{Partial}\;\mathrm{Eta}\;\mathrm{Squared}\;(\eta_{p}^{2})$
Stroke	3.485 × 10^5^	3.0	43.665	<0.001 ***	0.0278
Group	9.023 × 10^4^	1.0	33.918	<0.001 ***	0.0073
Stroke × Group (Interaction)	1.338 × 10^4^	3.0	16.766	<0.001 ***	0.0108
Residual	1.220 × 10^7^	4586.0	-	-	-
Average	1.277 × 10^7^	4593.0			

Note: *** indicates statistical significance at the *p* < 0.001 level.

**Table 6:** Optimized hyperparameters for the proposed CNN-BiLSTM architecture.

Hyperparameter	Value	Description
Optimizer	Adam	Adaptive Moment Estimation for robust convergence
Learning Rate	1 × 10^−3^	Initial step size for weight updates
Learning Rate Scheduler	StepLR	Gamma: 0.2, Step Size: 15 epochs
Weight Decay	5 × 10^−3^	L2 regularization to prevent overfitting
Loss Function	Cross-Entropy	Standard criterion for multi-class classification
Batch Size	64	Number of stroke samples processed per iteration
Max Epochs	50	Total training iterations (Early stability observed ~20)
Dropout Rate	0.5 (50%)	Dropout applied to the fully connected layer
Validation Strategy	5-Fold Stratified	Ensuring class balance across all folds

**Table 7:** Detailed classification performance categorized by tennis stroke techniques.

Stroke	Class	Precision	Recall	F1-Skoru	Accuracy
Forehand	Amateur	0.99	1.00	1.00	0.99
	Elite	1.00	0.99	0.99	
Backhand	Amateur	1.00	1.00	1.00	1.00
	Elite	1.00	1.00	1.00	
Service	Amateur	0.99	0.98	0.99	0.98
	Elite	0.97	0.98	0.98	
Volley	Amateur	0.99	0.99	0.99	0.99
	Elite	0.99	0.99	0.99	
General Average		0.99	0.99	0.99	%95.54

**Table 8:** Comparative analysis of the proposed framework with state-of-the-art studies.

Author (Year)	Experimental Paradigm and Classification Target	Feature Extraction and Data Processing	Best Performing Classifier	Performance (Accuracy %)
Gao & Zhang (2024) [19]	40 players (Elite vs. Amateur). IMU and pressure sensors on the racket. Skill level classification.	Acceleration and pressure variations, PCA-based dimension reduction.	SVM-PCA	94.08%
[24]	Runners. Wearable IMUs for biomechanical parameter estimation.	FFT, Time and Frequency domain features.	RF	R^2^: 0.97
[22]	Single IMU on the left wrist. Evaluation of 6 gymnastic movements.	Time-series to image transformation (GAF, RP, MTF).	ViTGS (Deep Learning)	99.04%
[27]	Smart racket (IMU + Piezoresistive). Detection of 5 stroke types.	Peak amplitude detection and Find-peak method.	Subspace k-NN	98.40%
[21]	36 players. Single IMU on the wrist. Stroke type and ITN level detection.	Time-domain features and StandardScaler/PCA.	SVM	90.00%
[10]	542 athletes. Optical data for Elite/Novice classification.	3D kinematic time-series, Ensemble feature selection.	RC and LSTM	77.90–79.00%
Proposed Study (2026)	39 players (11 Elite, 28 Amateur). Dual-wrist synchronized IMU fusion.	Automated feature filtering from raw signal patterns via Dynamic Windowing.	Hybrid CNN-BiLSTM Framework	95.54%

## Data Availability

The data presented in this study are available on request from the corresponding author. The data are not publicly available due to privacy restrictions regarding human participant biomechanical signals.
